# Gene Therapy Strategies Targeting Aging-Related Diseases

**DOI:** 10.14336/AD.2022.00725

**Published:** 2023-04-01

**Authors:** Jingyu Yu, Tianwen Li, Jianhong Zhu

**Affiliations:** Department of Neurosurgery, Huashan Hospital, Shanghai Medical College, Fudan University, National Center for Neurological Disorders, National Key Laboratory for Medical Neurobiology, Institutes of Brain Science, Shanghai Key Laboratory of Brain Function and Regeneration, Institute of Neurosurgery, MOE Frontiers Center for Brain Science, Shanghai, China.

**Keywords:** gene therapy, aging, aging-related disease, gene replacement, CRISPR

## Abstract

Rapid advancements have taken place in gene therapy technology. However, effective methods for treating aging- or age-related chronic diseases, which are often closely related to genes or even multiple genes, are still lacking. The path to developing cures is winding, while gene therapy that targets genes related to aging represents an exciting research direction with tremendous potential. Among aging-related genes, some candidates have been studied at different levels, from cell to organismal levels (e.g., mammalian models) with different methods, from overexpression to gene editing. The *TERT* and *APOE* have even entered the stage of clinical trials. Even those displaying only a preliminary association with diseases have potential applications. This article discusses the foundations and recent breakthroughs in the field of gene therapy, providing a summary of current mainstream strategies and gene therapy products with clinical and preclinical applications. Finally, we review representative target genes and their potential for treating aging or age-related diseases.

Aging is a natural process that involves the progressive decline of physiological functions and is a major risk factor for diseases such as neurodegenerative diseases, cardiovascular diseases, metabolic diseases, and malignant tumors. In 2013, López-Otin et al. proposed “The Hallmarks of Aging”. This paper gradually became the most widely cited reference in the field of aging. These hallmarks include primary markers of aging (genomic instability, telomere attrition, epigenetic alterations, and loss of proteostasis), antagonistic markers of aging (mitochondrial dysfunction, cellular senescence, and deregulated nutrient sensing) and integrative hallmarks (stem cell exhaustion and altered intercellular communication). In the early stage of aging, these three antagonistic markers provide protection and compensation for cells to withstand the four primary markers that trigger aging. However, as aging progresses, their compensatory ability is gradually lost, and the three antagonistic markers shift to promote aging, as indicated by 2 integrative hallmarks [1, 2]. These 9 hallmarks have provided support for the development of systematic treatment schemes for aging-related diseases.

As many aging or age-related diseases still lack effective treatment, therapies targeting aging have attracted attention at various stages and hallmarks of aging. For example, NAD^+^ plays a constructive role during the whole process of aging [3-8]; resveratrol induces mitophagy, regulates nutrient sensing and metabolism, and simultaneously upregulates sirtuin 1 (SIRT1) activity at the epigenetic level [9-12]; Senolytics selectively clear senescent cells that exhibit the senescence-associated secretory phenotype (SASP) [13-17]; and mTOR inhibitors (e.g., rapamycin and its analogs) induce autophagy [18-22] These strategies are currently at different stages of development, and their actual therapeutic efficacy as well as the adverse reactions are still far from satisfactory. Thus, these strategies are not widely used in clinical practice.

The progress of aging-related diseases often begins with alterations of a single gene or even multiple genes. Moreover, the efficacy of drugs frequently fades with progressing conditions, and unavoidable adverse reactions emerge with increased doses aiming to treat the progressing conditions. In the contrast, gene therapy provides hope that these diseases may be cured in the future because it principally focuses on the primary markers of aging. Gene therapy, or genetic therapy originally refers to approaches that treat genetic disorders by providing new DNA to specific cells or correcting the existing DNA [23]. In the 1960s, Nirenberg proposed genetic engineering applications that would allow human gene therapy. In the twenty-first century, various technologies of gene therapy have been created and developed, especially the clustered regularly interspaced short palindromic repeats (CRISPR) technology. These technologies allow not only editing, such as knockout, homologous recombination, and base editing, but also epigenetic modification (by altering protein expression without altering genetic information), such as repression, activation and demethylation of genes of interest, and other operations. These methods make it possible to regulate gene expression more concisely and precisely [24], providing more abundant applications for gene therapy. This article discusses the foundations of gene therapy as well as recent findings; it also reviews representative target genes and highlights their potential for treating aging or age-related diseases.

**Figure 1.**
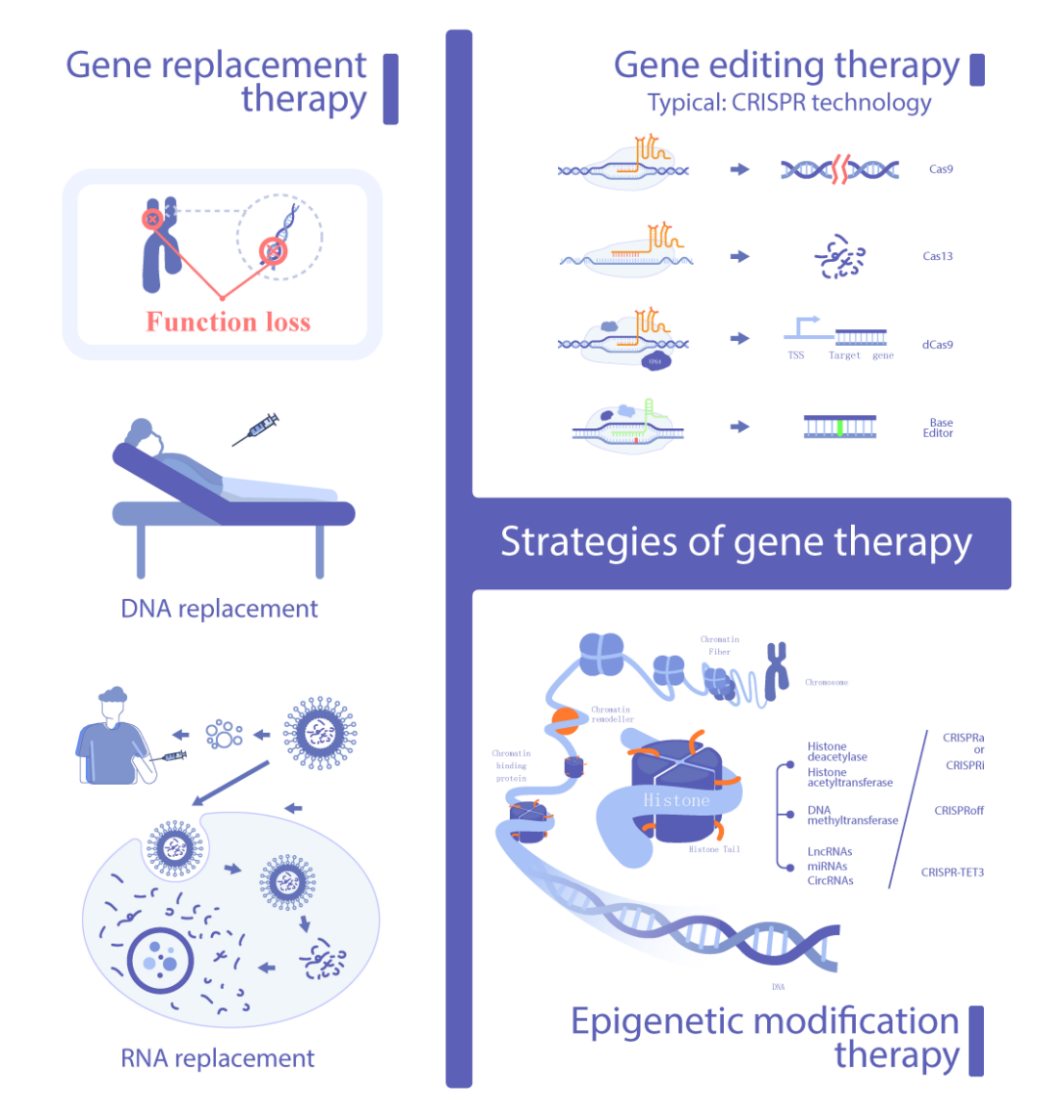
Strategies of gene therapy. The schematic enumerates the strategies of gene therapy summarized in this review.

## Strategies of gene therapy

Initially, gene therapy mainly referred to the introduction of an exogenous copy of complementary DNA (cDNA) into targeted tissues or cells to correct or compensate for defective endogenous genes [25]. With the advance in recognition and technology, gene therapy has shifted to focus on manipulating the expression of a certain gene [26] or modifying the genetic information responsible for a disease. Thus, gene therapy encompasses not only operations at the DNA level but also modification at the mRNA level, due to its ability to transmit genetic information.

Here, we summarize the current strategies according to the methods and level of manipulation of genetic information (Fig. 1).

### Gene replacement therapy

Gene replacement therapy, which was the original strategy of gene therapy, currently refers to replacing a pathogenic gene with a copy of a healthy gene or replacing a physiological gene with an engineered gene whose benefits have been reinforced. In this strategy, the most common delivery system is exogenous cDNA transfer mediated by viral vectors [27]. In 2003, the first commercialized gene therapy medicine, Gendicine (recombinant human p53 adenovirus) [28], which exerted a broad-spectrum anti-malignant tumor effect, was authorized in China. Since then, the therapeutic potential of various viral vectors has been expanded. Recombinant adeno-associated viruses (rAAVs) and lentiviruses are the most widely applied viral vectors. Although packaging capacity, immunogenicity and biosafety are always valid concerns, the appropriate and rigorous selection of alternative replacement routes (in vivo vs. ex vivo) and administration routes (systemic vs. local administration), as well as improvements in the tissue selectivity of the viral vectors often allow treatment without serious adverse reactions. Thus, in practice, the replacement mostly occurs at the functional level rather than accurately changing the genetic structure

Functional replacement at the RNA level, wherein therapeutic mRNA is mainly delivered through lipid nanoparticles, has flourished with the development of COVID-19 vaccines and is now a significant method for gene therapy. On the one hand, the delivered engineered mRNAs can be directly translated into functional therapeutic proteins that patients were originally unable to express. On the other hand, engineered mRNAs can express exogenous tools such as the CRISPR-Cas toolbox for implementing gene editing or modification [29]. The former approach is regarded as a safer method than direct genome engineering, given the short-lived nucleic acid activity of mRNAs; thus, it may be more flexible in vivo applications, while the latter approach provides more options for delivering therapeutic tools.

This strategy is preferable for conditions caused by a single gene defect, and it is the best for achieving local effects without complicated systemic interactions.

### Gene editing therapy

An alternative to overriding the expression of the disease-causing gene is to directly eliminate its effect by altering local specific DNA sequences to produce precise on-target mutations or small indels at the genomic level. These alterations include inserting a healthy or modified sequence and revising, removing or replacing the disease-causing sequence. This strategy represents the initial expectations for gene therapy. There are three powerful tools for genome editing: transcription activator-like effector nuclease (TALEN), zinc-finger nuclease (ZFN) and CRISPR. CRISPR technology is the most commonly used because of its availability, high efficiency and better extensibility. The broad category of CRISPR tools includes RNA-guided endonucleases that mainly induce double-strand breaks (DBS) and generates non-homologous end joining (NHEJ), microhomology-mediated end joining (MMEJ), and homology-directed repair (HDR). Of the endonucleases, Cas9 nucleases were the earliest applied to mammals and human cells, and Cas12 nucleases are mostly applied in nonmammalian cells because their protospacer adjacent motif (PAM) is rich in TT bases [30]. Cas13 nucleases can cleave single-stranded RNA, implementing the editing and regulation at the RNA level. Without causing strand breaks, base editors target conversions and prime editors produce exact substitutions, insertions and deletions [30]. In addition, adenosine deaminase acting on RNA (ADAR)-mediated RNA editing is another remarkable method currently under development that utilizes natural endogenous mechanisms [31].

Regarding clinical and preclinical applications, a limited number of registered and published studies have cautiously explored gene editing without the auxiliary cellular products in vivo, mostly using vehicles as well as local administration to battle high-grade tumor tissues [32, 33]. Although the publication of NTLA-2001 for Transthyretin Amyloidosis marked the first step toward in vivo CRISPR gene editing product [34], it was granted orphan drug by the United States Food and Drug Administration (FDA) and its success has limited expansibility. Since some DSB, indel and deletion induced by Cas9 were unexpected, it is generally unsafe to be straightforwardly performed *in vivo*, these uncertainties and ethical disputes must be resolved before further research can be conducted. Currently, gene editing is mostly implemented for developing products ex vivo [35] in case of dangerous off-target effects.

Despite the abovementioned results, it is worth noting that base editing which does not generate DNA strand breaks might present a safer option. Kathiresan et al. performed a near-complete knockdown of PCSK9 in the liver of cynomolgus monkeys with lipid nanoparticles (LNP)-encapsulated CRISPR base editors to lower cholesterol and thus treat atherosclerotic cardiovascular disease; they demonstrated efficient editing with mild off-target effect [36]. Recently, Monian et al. designed an efficient tool, namely, short chemically modified oligonucleotides called AIMers, that recruits endogenous ADAR without requiring exogenous enzymes and delivery vectors for adenine (A)-to-inosine (I) RNA base editing [31]. The stereopure chemically modified AIMers achieved effective editing in cynomolgus monkeys as well. The excellent examples of base editing may accelerate the development of in vivo gene editing products.

### Epigenetic modification therapy

Other prospective approaches to treat diseases involve modification at the genetic level without changing the nucleic acid sequences. These approaches alter the expression of genetic information, such as inactivating a disease-causing gene, activating antagonist genes of the disease-causing genes, adjusting the imbalance in gene expression or correcting abnormal chemical modifications [37-39]. With advances in the expanded CRISPR/Cas effector technology, tools, such as CRISPRa/CRISPRi (directly regulating gene expression [30]), CRISPRoff (initiating DNA methylation [40]), TET3 fused high-fidelity catalytically inactive Cas9 (dCas9) (demethylating methylated DNA [41]) and Cas13-directed methyltransferase (mediating efficient m6A modifications in endogenous RNA transcripts [42]), have been constructed and developed. These tools allow more diverse and delicate epigenetic modifications than those mediated by regulatory noncoding RNAs [e.g., short hairpin RNA (shRNA) and small interfering RNA (siRNA)][43]. Therefore, the operating procedures for epigenetic regulation are consequently trending toward standardization. This strategy is currently under development and has been mostly tested for treating malignant tumors and CRISPR screening in vivo [37-39].

## Gene therapy products with clinical and preclinical applications

According to the FDA's 2018 classification, there are 5 main categories of gene therapy products [26]. However, not all of these 5 categories are suitable for wide applications in clinical practice; In addition, other novel products are qualified for direct delivery and exhibit substantial promise (Table 1).

### Classic viral vectors

**Recombinant adeno-associated viruses (rAAV)** are currently the most frequently applied vector in interventional clinical trials of gene transduction. An rAAV consists of a nonenveloped protein containing a linear single-stranded DNA sequence. Compared with adenovirus, retrovirus and lentivirus vectors, the packaging capacity of rAAV vectors is small; Multiple or hybrid vectors are necessary to transduce genes of interest. However, rAAVs do have relatively high safety, low immunogenicity, and efficient transduction in a wide range of target tissues. Even if the loaded exogenous gene cannot be inserted into the genome of the host, it can be stably expressed in vivo as a satellite for a long duration [44].

With different serotypes (serotypes 1-12) and synthetic serotypes of viral capsid proteins, rAAV vectors can be designed to target specific cells or tissues by an appropriate route of administration [45]. Among these serotypes, vectors based on serotype AAV2 are the most common delivery platforms. Other platforms are also under development. For example, serotypes AAV9 and AAVRh. 10 can cross the blood-brain barrier (BBB) and mediate the expression of transduced genes in neurons and gliocytes via intravenous infusion [46]. Currently, rAAV vectors have been successfully applied to alleviate the following diseases: hemophilia, hereditary eye diseases, and some neurodegenerative diseases [47-49].

**Lentiviral vectors (LVs)** are modified from human immunodeficiency virus (HIV); during modification, their ability to infect, replicate and cause diseases was reduced, while the expression of the transferred plasmid was enhanced [50]. Compared with rAAVs, LVs insert exogenous DNA into the genome, probably leading to genomic mutation, and have higher immunogenicity. But LVs are capable of loading larger sequences and are suitable to be employed in overexpression of large protein and CRISPR screens [44]. Nevertheless, its application of genetic modification for cellular products (i.e., stem cells and immunocytes) ex vivo or via local administration, such as intracerebroventricular injection and stereotactic injection, in immunologically privileged sites has resulted in potent efficacy [48, 50]. Presently, LVs are well-performed in clinical practice of primary immuno-deficiency diseases, hemoglobinopathies and clinical trials of neurodegenerative diseases [50].

Aside from their primary role as vectors for gene replacement therapy, viral vectors can also deliver tools, including the CRISPR system and noncoding RNAs. Therefore, viruses play indispensable and multiple roles in gene therapy.

**Table 1:** Comparison of the principles and characteristics of various gene therapy products with clinical and preclinical applications.

Types	Principles	Immunogenicity	Targetability	Reproducibility	Maneuverability
Viral vectors	Expressing exogenous genes intracellularly in a particular tissue through infection.	Compared with adenovirus, retrovirus and lentivirus vectors, rAAVs vectors have relatively low immunogenicity, but still cannot be underestimated in clinical trials for components such as capsid protein and bacteria-derived Cas9 protein [55, 167].	Prolonged expression of DNA-encoding editing agents increases the incidence of off-target mutagenesis [61].	Low. A second injection most probably leads to be neutralized by acquired immunity [55, 168].	Modular assembly.
Lipid nanoparticle	A stable nanostructure in which nucleic acid molecules can be encapsulated in the interior core through electrostatic interactions with the lipids by rapid mixing[ 52].	Relatively low [55]. Widely used coated PEG lowering LNP self-aggregation, opsonization, or phagocytosis. Increasing the time interval between injections alleviates the accelerated blood clearance phenomenon [169].	Easily gathered in the liver but can be mediated for tissue or cell-specific delivery by modifying and optimizing, such as coated with antibodies, additional surface ligands or chemically modified [52, 170].	High[97].	The trend to rely on commercial manufacturing.
Virus-like particles	Self-assembling, mimicking the structure of a virus particle and being uptaken by receptor-mediated endocytosis but not replicating [58].	Poorly tested. Could be up to the used virus structure.	Reporting a comparable on-target editing but minimal off-target effect compared to rAAV approaches in vivo [61]. More specific targeting can be achieved by displaying a chemically or genetically modified receptor-binding domain on the VLP surface[58].	Poorly tested.	Modular assembly.
Extracellular vesicles	Endogenous vesicles mediating intercellular communication through transferring RNA and protein [171].	Low [172]. Recommended using EVs in an autologous manner [173].	May have an inherent preferential targeting ability and constitute a natural route for efficient transport [173].	Reporting no elicit visual signs of toxicity the in repeated administration of EVs in mice [172].	Traditionally purified by ultracentrifugation [174].
Genetically modified cellular products	Modified human cells replace the damaged original cells or play a stronger immune role.	Poorly understood. Associated with the cytokine-release syndrome, immune effector cell-associated neurotoxicity syndrome and other immune-mediated adverse events[175].	On-target off-tumor effects relatively occur more in the exploration of solid tumors[77].	Suboptimal [175]. May be related to antigen escape [77].	Often using a viral vector.

### Novel vehicles

Despite the strategy-optimized classic viral vectors as well as cellular products, more convenient and reproducible products are constantly being explored.

**Lipid nanoparticle (LNP)** systems are pioneering nonbiological or chemical gene therapy products enabling direct delivery of modifying tools. The production process of LNP is simple and standardized without concern for biosafety [51]. The popularity of this technology, which is the main delivery platform for the COVID-19 mRNA vaccine, has risen rapidly in the past two years [52]. Long before research on the COVID-19 mRNA vaccine was published, LNP systems had already shown promise for genetic medicine, specifically, siRNA, mRNA and plasmids [53]. Nowadays, their availability is increased in the field of gene therapy. Recently, Rizvi et al. have established a transiently modified mRNA-LNP expressing hepatocyte-growth-factor (HGF) and epidermal-growth-factor (EGF) in hepatocytes. These LNPs effectively induced liver regeneration in mouse models of chronic nonalcoholic fatty liver injury and an acetaminophen-induced acute liver injury [54]. Kenjo et al. developed a pH-dependent ionizable lipid; together with Cas9 mRNA and single-guide RNA (sgRNA), it was fashioned into an LNP that selectively targeted skeletal muscle. It induced steady genomic exon skipping and restored dystrophin proteins in a mouse model of Duchenne muscular dystrophy [55]. Compared with rAAV injections, LNP showed lower immunogenicity even from repeated intramuscular injections. Additionally, Rurik et al. generated a transiently antifibrotic chimeric antigen receptor (CAR) structure encoded by modified mRNA delivered by CD5-targeted LNPs to produce CAR T-cells in vivo, alleviating the fibrosis and deterioration of cardiac function caused by increased cardiac afterload in a mouse model [56]. Moreover, the analog liposomal nanoparticle [57] can also be developed. Although the efficiency, targeting capabilities and reproducibility of LNPs have been improved, as seen in the studies above, there is still room for improvement and expansion to treat other pathological processes.

**Gold nanoparticles (AuNPs)** have also been reported as a prospective vector to deliver CRISPR tools in vivo, but they are still in an early stage of development [51].

**Virus-like particles (VLPs)** are self-assembling structures that closely mimic the form and size of a virus particle but lack the ability to replicate due to not carrying genetic material [58]. VLPs are a prospective candidate tool for vaccines as well as currently considered a potential delivery vehicle in the field of gene therapy [58]. For gene therapy, current research on VLPs focuses on the selection of referenced viral capsids and the optimization of enrichment strategies. Recently, Ling et al. developed a system named mLP-CRISPR, in which mRNA-carrying lentiviral particles delivered mRNAs encoding SpCas9 and guide RNA (gRNA) simultaneously by a modular operation similar to the classic virus packaging process. Applications of mLP-CRISPR that targeted vascular endothelial growth factor A (Vegfa) in a mouse model of age-related wet macular degeneration reduced Vegfa expression by 44% and reduced choroidal neo-vascularization after a single subretinal injection [59]. Given this finding, Yin et al. directly targeted the genome of herpes simplex virus 1 (HSV-1) using a strategy with mLP-CRISPR (designated HSV-1-erasing lentiviral particles; HELP). This study clearly illustrated the high therapeutic potential of CRISPR for treating infectious diseases by targeting the genome of the virus [60]. Although the modified VLPs show good efficacy for treating diseases of the bulbus oculi, their targetability, immunogenicity and reproducibility have yet to be addressed as the immune mechanism of the eye is different from other organs. To optimize the efficiency of delivery tools in vivo, Banskota et al. used the strategy of ribonucleoproteins (RNPs) to conduct gene editing. After a few generations of screening, they recommended fourth generation engineered virus-like particles (eVLPs) with more than 50% knockdown of the target and no detected off-target effects from a single injection [61].

**Extracellular vehicles (EVs)** have been applied to deliver CRISPR tools in vitro and drugs in vivo. Most past studies focusing on EVs with gene therapy involved siRNAs and microRNA (miRNAs) [62]. At present, more approaches, such as the Cas9-sgRNA that employed RNPs (see the VLP section), have been described to deliver the CRISPR tools in vivo [63]. The transience of EVs for CRISPR effectors, especially genome editing effectors, is an advantage as it reduces the likelihood of off-target and immune responses [63]. However, it also introduces issues with efficacy and efficiency. During early development, Kim et al. reported exosomal delivery of CRISPR/Cas9 in vivo derived from SKOV3 cancer cells. Compared to control exosomes derived from HEK293 epithelial cell line, experimental exosomes derived from SKOV3 cancer cells carrying CRISPR/Cas9 not only allowed efficient inhibition of poly (ADP-ribose) polymerase-1 (PARP-1)-induced apoptosis in ovarian cancer but also preferentially accumulated in the lesion in a SKOV3 xenograft mouse model, providing tropism-dependent targeting [64]. To improve the efficiency of EV delivery, Yao et al. developed an actively enriching mechanism utilizing interactions between the RNA aptamer and aptamer-binding protein (ABP). This approach provided an upgraded strategy based on RNPs. They modified the sgRNA with an RNA aptamer com and modified both termini of the tetraspan protein CD63 that is characteristically expressed on the surface of exosomes with the com-binding ABP Com so that the com-modified Cas9-sgRNA RNP would be enriched by CD63-Com fusion protein by Com/com interaction. After verifying the in vitro results, they further observed high dystrophin expression in del52hDMD/mdx mice through EV-delivered DMD exon 53-targeting RNPs [63]. In addition, other smart modifications have been proposed to make EVs more available for gene therapies [65-68].

In 2021, Zhang et al. developed a brand-new mRNA delivery system named selective endogenous encapsidation for cellular delivery (SEND). It was implemented by modularly engineering PEG10 cotransfected with cargo RNA and fusogen plasmids to package, secrete, and deliver [69]. Although they did not display any in vivo results in their publication, PEG10 (a mammalian homolog of capsid protein screened out in both mice and humans) exhibits high histocompatibility and low immunogenicity. Therefore, SEND may present a deep impression in vivo application soon.

### Genetically modified cellular products

Genetically modified cellular products are deemed relatively more stable than viral vectors. The origin of these cells can be autogenous or allogeneic, but the applicability of autogenous products is wider than allogeneic products due to histocompatibility. As this approach originated with bone marrow transplantation technology, prior studies that modified the genome of cells ex vivo were concentrated on peripheral hemolymph diseases. In 2017, the FDA approved CAR T-cells, a cellular product genetically modified [70] to boost its effect on hemolymph tumors, making another milestone in gene therapy. Subsequently, CAR technology has been developed involving natural killer cells (NK cells), natural killer T cells (NKT cells), macrophages [71-73] and other immune cell modifications, even combined with CRISPR- editing [74] and targeting other characteristic molecular markers.

The cells genetically modified into cellular products are principally comprised of immune cells and stem cells. A representative of modified immune cells is CAR T. CAR T treatment not only provided more clinical options for treating hemolymph tumors but also opened the door to targeting solid tumors in the context of precision medicine [75-77]. Theoretically, the presence of characteristic molecular markers can direct CAR structure to multiple pathologic processes in addition to the tumors. For example, CAR T targeting fibroblast activation protein (FAP) has been designed to treat anti-myocardial fibrosis [56]. Soon, CAR structure may even be directed to characteristic molecular markers of aging to treat aging-related diseases. Modifications to stem cells have focused chiefly on hematopoietic stem cells (HSCs) and mesenchymal stem cells (MSCs). In hemoglobinopathies, pathological hemoglobin can be replaced by normal hemoglobin differentiated from ex vivo gene-corrected autogenous HSCs, solving the tissue-matching issues [50]. For instance, HEMACORD® and other cord blood stem cell products have been successfully applied in the clinic. For malignant tumors, MSCs exhibit tolerable histocompatibility, low immunogenicity and are easily obtained; their capacity of homing and migrating to the tumor and their natural antitumor properties have been demonstrated in multiple animal models [78]. Engineering can further enhance their ability to target specific tumors. A recent preclinical study utilized engineered allogeneic MSCs as sequential therapy after the resection of glioblastoma (GBM) [79]. These MSCs were engineered by LV infection and designed to conditionally express TNF-related apoptosis inducing ligand (TRAIL), which is a ligand of CD146, to increase the orientation and tropism of MSCs to GBM. Next, the clinical perioperative process, preparation process and intraoperative intratumoral administration are simulated on GBM-derived xenografts of mice. The survival of these mice was prolonged in this study, demonstrating the safety and efficacy of engineered MSC therapy. In addition, the other sources of stem cells, involving personalized induced pluripotent stem cells (iPSCs) such as harvested from a skin biopsy, may gradually step into the clinical [80].

Ex vivo operations on cells (often using a viral vector, mostly LVs) have allowed for bolder modifications while replacing originally damaged cells or strengthening their immune role. In light of the inherent characteristics of immune cells and stem cells, genetically modified cellular products may allow the clearance and replacement of senescent cells. However, the safety and efficacy of these products for wider application remain largely unknown.

## Applications of anti-aging gene therapy

**Figure 2.**
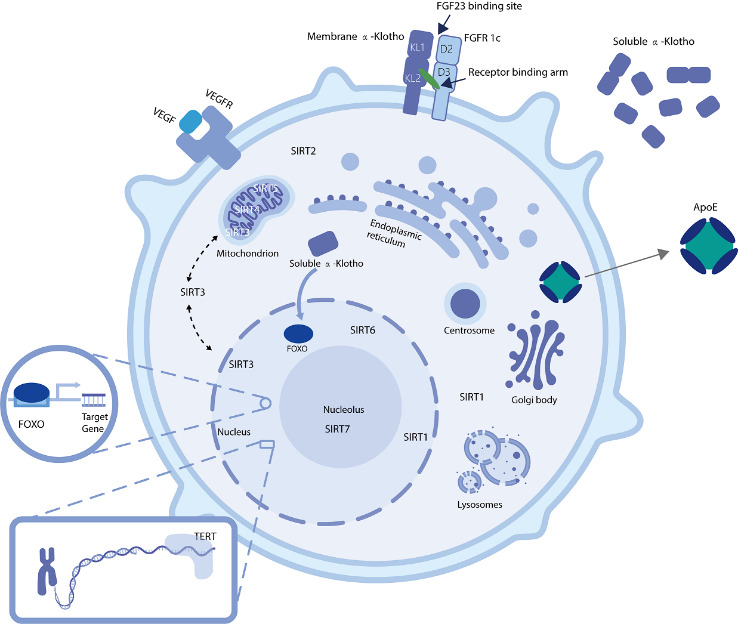
Schematic of the cellular components of proteins encoded by representative aging-related genes.

As research on aging has progressed and sequencing technologies have improved [81-86], an increasing number of aging-related genes have been identified. Detailed comparisons among diverse groups of tissues and cells in different phases and spaces have been conducted. There may be millions of age-related genes that will be identified in the future [87], exhibiting positive and negative correlations with aging. Furthermore, some studies have reported overlap in the spectra of age- or aging-related diseases and aging-related genes [87], which suggests that developing aging-related genes as therapeutic targets may have great potential (Fig. 2).

### Targets of aging-related genes for clinical applications

Telomerase gene. Telomeres are structures at the ends of chromosomes that are essential for maintaining the length of telomeric DNA and preserving chromosomal stability. In human chromosomes, the DNA sequences of telomeres are noncoding 5'-TTAGGG-3' repeat sequences, approximately 15-20kb long; the telomeres are maintained by de novo addition from telomerase [88]. The telomerase reverse transcriptase (*TERT*) gene encodes the rate-limiting catalytic TERT protein, a subunit of telomerase [89, 90]. Studies on telomeres and telomerase have been conducted since the start of research on aging. Many studies have shown that defects in telomeres or telomerase exert a substantial influence on the development of aging-related diseases [91-96]. The current advanced understanding of TERT allowed us to target the *TERT* gene to treat human diseases. In 1999, Bodnar and his team transduced different TERT-negative human endothelial cells with myeloproliferative sarcoma virus or plasmid vector carrying *hTERT* in vitro. The difference between the negative control clones and those expressing *hTERT* provided solid early evidence of the effects of *TERT* in delaying aging and elongating telomeres while maintaining a normal karyotype [97]. In subsequent studies on other cell types with similar methods, the high activity and expanded replicative potential of telomerase promoted further exploration.

Some in vivo experiments have already reported that *TERT* gene therapy exhibits exciting efficacy for treating diverse diseases. Maria A. Blasco. and her team have made great strides in the exploration of different diseases in mouse models via gene replacement (Table 2). In their earlier research, Bernardes de Jesus et al. tentatively introduced AAV-mouse *Tert* into 12- and 24-month-old mice, and they found noticeable improvements in various aging-related molecular biomarkers. Interestingly, an increase in median lifespan was also observed compared to the catalytically inactive *TERT* control group[98]. Later, Bar et al. designed an AAV9 vector that expressed *Tert* in cardio to treat heart failure after myocardial infarction (MI). Intravenous injection of this vector into mouse models of myocardial infarction showed that mice with the vector expressing *TERT* had less damage to the cardiac indices of both structure and function, decreased mortality and improved biomarkers [99]. In a follow-up study, Bar et al. concluded that high doses of AAV9-*Tert* could act on the bone marrow in two different mouse models of short telomere-induced aplastic anemia (*Trf1*- and *Tert*-deficient mice). Specifically, it appeared that bone marrow hematopoietic stem cells regained their functions, the aplastic phenotype was rescued, and the lengths of telomeres in both peripheral blood and bone marrow cells, blood counts, and survival were all improved [100]. Subsequently, Povedano et al. explored *TERT* gene therapy through a similar method in a mouse model of low-dose bleomycin-induced pulmonary fibrosis [101]. Whittemore et al. also applied it in a mouse model of neurodegeneration due to short telomeres [102]. Both of these models reflected optimistic phenotypic improvements. In addition, studies have reported an increase in the expression level of the *TERT* gene through telomerase activator (TA) which exerts a therapeutic effect. For instance, Wan et al. administrated dietary powdered TA-65 to a transgenic mouse model overexpressing human wild-type α-synuclein which induced Parkinson's disease and observed increased expression of *TERT*, improved motor function and increased autophagy [103]. Moreover, no malignant tumor tendency was observed in the above studies when the expression of *TERT* increased, which serves as a basis for further exploration and advances in clinical trials.

**Table 2:** Representative anti-aging mammalian experiments of gene replacement therapy

Targeting genes	Conditions	Titles	Authors	Models	Methods	Primary measure(s)
*TERT*	Myocardial infarction	Telomerase expression confers cardioprotection in the adult mouse heart after acute myocardial infarction	Bar et al., 2014	A mouse model of myocardial infarction induced by coronary artery ligation	Intravenously injecting AAV9-*Tert* specifically to the heart	Mortality; cardiac morphology and function; telomere length; the number of cycling cardiomyocytes
	Aplastic anemia	Telomerase gene therapy rescues telomere length, bone marrow aplasia, and survival in mice with aplastic anemia	Bar et al., 2016	2 mouse models of short telomere-induced aplastic anemia (*Trf1*- and *Tert*-deficient mice)	Intravenously injecting AAV9-*Tert*	Mortality; telomere length in blood and bone marrow; blood counts
	Pulmonary fibrosis	Therapeutic effects of telomerase in mice with pulmonary fibrosis induced by damage to the lungs and short telomeres	Povedano et al., 2018	A mouse model of pulmonary fibrosis owing to a low-dose bleomycin insult and short telomeres	Intravenously injecting AAV9 -*Tert* preferentially targets regenerative alveolar type II cells (ATII)	Percentage of cells positive for γH2AX, reduction in the abundance of p21 and p53-positive cells, decrease in caspase 3-positive cells in fibrotic lungs; proliferation of ATII cells; gene expression changes
	Neurodegeneration	Telomerase gene therapy ameliorates the effects of neurodegeneration associated with short telomeres in mice	Whittemore et al., 2018	A telomerase-deficient *Tert^-/-^* mouse model	Intravenously injecting AAV9-*Tert*	Percentage of cells positive for γH2AX marker in the hippocampus, the dentate gyrus, and the neocortex and the level of Trp53 mRNA in the brain; the intensity of tyrosine hydroxylase fluorescence in dopaminergic neurons; levels of cells positive for GFAP in the hippocampus, dentate gyrus, and neocortex; the Barnes maze test.
*KL*	Alzheimer's Disease	Lentiviral vector-mediated overexpression of Klotho in the brain improves Alzheimer's disease-like pathology and cognitive deficits in mice	Zeng et al., 2019	The APP/PS1 transgenic mice of Alzheimer's disease	Injecting intracerebroventricularly the lentiviral vector that encoded the transmembrane full-length form of mouse Klotho cDNA	Compact amyloid plaques and Aβ1-42-positive amyloid plaques in the brain; neuronal loss in the cerebral cortex; reductions of synaptophysin expression in the hippocampus and cortex; vascular pericyte coverage by immunostaining with platelet-derived growth factor receptor β and Lycopersicon esculentum lectin and measured cerebral blood flow in the mouse cerebral cortex; autophagy.
	Alzheimer's Disease	Klotho overexpression improves amyloid-β clearance and cognition in the APP/PS1 mouse model of Alzheimer's disease	Zhao et al., 2020	The APP/PS1 mouse model of Alzheimer's disease	Injecting lentivirus that carried full-length mouse Klotho cDNA in the lateral ventricle of the brain	The levels of soluble and insoluble Aβ_1-40_ and Aβ_1-42_ in the brain and serum; neuronal loss in the hippocampal CA1 area and cortex; activation of the NLRP3/caspase-1 signaling pathway; The mRNA and protein levels of LRP1 and P-gp, ABCA1 and RAGE in lectin-positive endothelial cells in the cortex and choroid plexus.
*SIRT*	Hutchinson-Gilford progeria syndrome	Vascular endothelium-targeted *Sirt7* gene therapy rejuvenates blood vessels and extends life span in a Hutchinson-Gilford progeria model	Sun et al., 2020	The Lmna^f/f^; TC mice with progerin expression induced by Tie2-Cre.	On-site injection of AAV1 cassette with *Sirt7* gene expression driven by a synthetic ICAM2 promoter	The indices of neovascularization, aging features, and life span.

In addition to *TERT* gene replacement, the expression level of endogenous *TERT*/telomerase can be relatively precisely regulated through modification with the widely used CRISPR technology (Table 3). These studies now mainly focus on malignant tumors, mostly when considering patient safety. Abnormal activation of *TERT*/telomerase that escapes telomere attrition and cellular senescence is fundamental to cell immortalization and malignant transformation [104, 105]. Thus, inhibiting *TERT*/telomerase in tumor cells [106] or promoting the expression of its antagonist are reasonably effective treatment designs. Dai *et al.* introduced a telomerase-activating gene expression (Tage) system constituted of an artificial transcription factor of telomeric repeats and an effector telomerase-activating Cas9, both of which were delivered by rAAV. This system effectively cut the abnormal telomeres and caused DNA damage in malignant tumor cells but left normal tissues unaffected *in vitro* and *in vivo* [107]. Li et al. fused adenine base editors to inactivate Campylobacter jejuni CRISPR/Cas9. Through sgRNA-guided single-base editing, the C>T mutation of the *TERT* promoter, -124 upstream of the start codons, was corrected to C. This correction blocked the members of the E26 transcription factor family from binding, suppressing the abnormal activation of the *TERT* promoter and ultimately inducing senescence and proliferation arrest in glioblastoma [32]. In addition, there are reports of anti-*TERT* antisense oligonucleotide (ASO) treatment [108].

**Table 3:** Representative anti-aging mammalian experiments of gene therapy through the CRISPR technology modifications.

Targeting	Conditions	Titles	Authors	Models	Methods	Strategies	Primary measure(s)
Telomere repeat sequences	Cancer	Cancer therapy with a CRISPR-assisted telomerase-activating gene expression system	Dai et al., 2019	The tumor-bearing mice with cancer cell Hepa1-6	A single-dose intravenous administration of a developed HO-based Tage system by using AAV as gene vectors	Gene editing	mean tumor size
The -124C>T *TERT* promoter mutation	Glioblastoma	Programmable base editing of mutated *TERT* promoter inhibits brain tumor growth	Li et al., 2020	The GBM cells (2 × 105) in 5 μl DMEM were injected intracranially into four-week-old female athymic nude mice.	AAVs expressing sgRNA-guided and catalytically impaired Campylobacter jejuni CRISPR-associated protein 9-fused adenine base editor (CjABE)	Gene editing(Base editing)	survival times; correction rates of the DNA region spanning the -124C>T mutation in the *TERT* promoter of the combined tumor tissues; telomere length; expression levels of TERT, Ki67 and cleaved PARP1; cell proliferation assay.
Hyper-methylated Klotho promoter	Renal fibrosis	High-fidelity CRISPR/Cas9- based gene-specific hydroxymethylation rescues gene expression and attenuates renal fibrosis	Xu et al., 2018	The Unilateral ureteral obstruction renal injury murine model	Intrarenal artery/vein infusion with Lentivirus expressing dHFCas9-TET3CD-*Kl*-sgRNA	Epigenetic modification	*KL* promoter methylation level; percentage of total interstitial fibrosis and immunostained positive cells in each group

In telomerase gene therapy, the effects of activation and inhibition are seemingly contradictory but reflect the temporal and spatial specificity of gene expression. Telomerase is relatively silent in most normal somatic cells but is activated in 90% of cancer cells; thus, therapeutic strategies aim to activate telomerase expression in normal somatic cells and inhibit it in malignant cells. However, this generates stricter requirements on targeting in designs for therapeutic strategies.

After conducting preliminary experiments with mammals, some researchers have commenced clinical trials (Table 4). A drug called LGT, an AAV-*hTERT* vector, was tested in 3 diverse directions in Phase I clinical trials in 2019: 1) targeting aging by intravenous administration (NCT04133649) [109], 2) targeting Alzheimer's disease by intravenous administration combined with intrathecal injection (NCT04133454) [110], and 3) targeting critical limb ischemia by intravenous administration (NCT04110964) [111]. These clinical trials were expected to be completed by January 2021, but no data have been published yet.

*APOE*. *APOE* is a gene currently recognized to have a robust association with human longevity [112]. Apolipoprotein E (APOE) protein is a pivotal regulator of lipid metabolism and is abundantly distributed in the liver, kidney, fat, and brain [113]. The ε4 allele significantly negatively predicts longevity, while the ε2ε2 or ε2ε3 genotypes significantly positively predict longevity[112]. Moreover, in the light of multiple large-scale genome-wide association analysis (GWAS) and genome-wide association meta-analyses, the *APOE* ε4 allele is the strongest genetic risk factor for sporadic Alzheimer's disease (AD), while the *APOE* ε2 allele is the strongest hereditary protective factor. A recent study reported that APOE functions as a destabilizer for heterochromatin in the mediation of cellular senescence [114]. However, its mechanistic role in aging and AD is far from clear. Present studies on the relationship of APOE to the pathogenesis of Alzheimer's disease have involved β-amyloid (Aβ), tau neurofibrillary degeneration, microglia, astrocyte responses and the BBB [115, 116]. These pathological processes are strongly associated with cognitive impairment, which may lead to a therapeutic target in the future. The therapeutic application of APOE is far more varied in mouse models of human APOE allele compared with the genes and their encoding products described above, including increasing or decreasing the expression of APOE, enhancing its lipidation, and blocking the interactions between APOE and Aβ [117, 118]. Nevertheless, these methods are no substitute for the successful switching of APOE alleles by gene therapy strategies in neurons and glial cells [119]. The alteration of isoforms from *APOE* ε4 to *APOE* ε2 by AAVrh.10h *APOE* ε2 vectors has been studied in a phase I clinical trial (NCT03634007) [120] (Table 4). To date, more detailed studies of its mechanisms, the associations with other aging-related diseases, and the application of CRISPR/Cas9 editing technology in mouse models remain highly important.

### Potential targets of the aging-related genes for clinical applications

*KL*, *KLB* and *FGF* family. *KL*, another classic aging-related gene, has a much shorter research history than that of the TERT gene, just over twenty years. In 1997, Japanese scientists Kuro-o *et al*. reported mutant mice with this gene deficiency. These mice exhibited a phenotype similar to human premature aging with degeneration and failure of multiple organs [121], but rescuing the *KL* delayed their aging and extended their lifespan up to 30% [122]. Continued research revealed that Klotho proteins encoded by *KL* and *KLB* are a family of proteins, including α-Klotho and its homolog β-Klotho, which are essential components of the endocrine fibroblast growth factor (EGF) receptor (FGFR) complex, ensuring high affinity when the corresponding type of FGF binds. The *KLB* that encodes β-Klotho is mainly expressed in adipocytes, liver, testis, and stomach, but we poorly understand this gene or its protein. The *KL* encodes α-Klotho, and they are the aging-related genes and proteins that we generally refer to when we discuss klotho. The expression of *KL* is especially high in the kidneys. It can be transcribed into two different transcripts. One of them is translated and expressed on the cell membrane (m-KL). This is the main form that interacts with FGF23 and FGFR to regulate phosphate homeostasis. Defects in m-KL can lead to not only chronic kidney disease (CKD)-related phenotypes but also a multisystem aging syndrome involving the central nervous system, respiratory system, reproductive system and other systems [123-125]. m-KLs can be cleaved off by the metalloproteases Adam 10 or Adam 17, producing a different soluble form (s-KL). The other transcript is translated into another soluble form with a characteristic C-terminal sequence and is secreted extracellularly [126, 127]. s-KLs play a complex role in humoral and even long-distance endocrine processes through a variety of receptors and signaling pathways including inhibiting the insulin/IGF-1 signaling pathway, WNT signaling pathway, p53/p21, increasing the level of forkhead box subgroup O (FOXO) phosphorylation, and mediating the phosphorus and calcium metabolism, as well as exerting different anti-aging effects including anti-oxidative stress, anti-cell senescence, promoting autophagy, anti-exhaustion of stem cells, etc.[128, 129]. In addition, other researchers have claimed that FGF may be the core of anti-aging effects in klotho [130]. Kuro-o et al. also summarized their effects as the "FGF-Klotho protein endocrine axis" [123].

**Table 4:** Existing interventional clinical trials of aging-related genes for aging- or age-related diseases.

Conditions	ClinicalTrials.gov Identifier	Vectors	Routes of administration	Doses	Primary outcome measure(s)	Time to follow-up	Adversereactions	Status
Aging	NCT04133649	AAV-*hTERT*	intravenous injection	a single IV dose	Incidence of serious adverse events and adverse events throughout the study	12 months	Not yet reported	Unknown
Alzheimer Disease	NCT04133454	AAV-*hTERT*	intravenous injection and intrathecal injection	a single dose	Incidence of serious adverse events and adverse events throughout the study	12 months	Not yet reported	Unknown
Early Onset Alzheimer's Disease	NCT03634007	AAVrh.10h-*APOE* ε2(LX1001)	CNS Administered	5.0 × 10^10^ gc/mL CSF;1.6 ×10^11^ gc/mL CSF;5.0×10^11^ gc/mL CSF	1. Proportion of participants with treatment-emergent adverse events and serious adverse events; 2. Proportion of participants with treatment-emergent AEs and SAEs at each dosage.	1 year	Not yet reported	Recruiting

Exploring concrete mammalian models (mostly mouse models) of aging-related diseases has revealed the therapeutic effect of enhancing *KL* expression in neurodegenerative diseases, chronic kidney diseases, cardiovascular diseases, etc.

Earlier research on the *KL* explored its role in neurodegenerative diseases. Dubal et al. crossed *KL*-overexpressing transgenic mice with human amyloid precursor protein (hAPP)-J20 transgenic AD mice. Among their offspring, those expressed hAPP and Klotho exhibited reductions in the rate of premature mortality, alleviated network dysfunction and increased postsynaptic density of the learning and memory-associated N-methyl-D-aspartate receptor (NMDAR) GLUN2B subunit [131]. Zeng and Zhao et al. intracerebroventricularly injected lentiviruses encoding Klotho into amyloid precursor protein/presenilin 1 (APP/PS1) transgenic mouse models of AD and observed Aβ clearance by autophagy and amelioration of cognition in mice [132] (Table 2). Their detailed investigation of mechanisms of various cellular phenotypes, including cognitive function, Aβ-related neuropathology and microglial transformation. And the Aβ transport was conducted with lentiviruses carrying full-length mouse Klotho cDNA [133], further supporting the *KL* as a therapeutic target.

In studies of kidney disease, Xu et al. fused the catalytic domain of TET3 (catalyzing the hydroxymethylation of methylated DNA) to high-fidelity inactive Cas9 (dHFCas9) and administrated the combination to a mouse model of unilateral ureteral obstruction (UUO) renal injury (Table 3). Under the guidance of Rasal1-sgRNA and *KL*-sgRNA, it rescued gene expression and alleviated renal fibrosis, which directly demonstrated the role of the *KL* gene in the maintenance of kidney function [41].

Studies on applications in cardiovascular diseases have not progressed as far as those in neurodegenerative diseases and kidney diseases. Olejnik et al. successively explored the effect of Klotho on ischemia-reperfusion injury in vitro in human cardiomyocytes and rat Langendorff isolated heart perfusion models. Treatment with klotho protein promoted cardiomyocyte viability and metabolic activity, supporting klotho and *KL* as a feasible direction of therapy in some cardiovascular diseases [134]. In addition, some studies have also indirectly indicated the potential role of Klotho protein in aging-related diseases. For example, by enhancing the interaction of FGF21with its coreceptor β-Klotho via an AAV-FGF21 vector that triggered the ERK1/2 and MAPKs signaling pathways to combat metabolic diseases [135, 136].

Preclinical evidence has demonstrated that Klotho has broad therapeutic promise for treating various aging-related diseases. However, no interventional clinical trials have been conducted to assess the clinical potential of *KL* and Klotho.

*FOXOs*. The effect of the *FOXO* gene on longevity was discovered in an analysis comparing long-lived Japanese individuals (95-100 years old) and typical older individuals (death before the age of 81) [137]. The forkhead box sub-group O (FOXO) protein family is a family of transcription factors that are activated under stressful circumstances, including starvation, energy deficiency, oxidative stress, and DNA damage; these transcription factors then promote posttranslational modifications such as phosphorylation, monou-biquitination, methylation and glycosylation [138], regulating downstream genes involved in the cell cycle, cell death, autophagy, cell metabolism, cellular antioxidants, etc.[139, 140]. Among the *FOXO* family members, *FOXO1*, *FOXO3*, *FOXO4*, and *FOXO6* are found in mammals [139]. In neural stem cells, FOXOs are essential for maintaining a dormant state and clearing reactive oxygen species (ROS); in mature neurons, the transcriptional activity of FOXOs is critical for protecting axons from age-dependent degeneration [138, 141]. Research on neurodegenerative diseases suggests that the downregulation of *FOXO* expression is closely related to impairment in cognitive and motor functions and contributes to the progression of Alzheimer's disease, Parkinson's disease, amyotrophic lateral sclerosis, Huntington's disease, etc. [138, 140, 141]. In malignant tumors, although FOXOs mainly exert antitumorigenic effects [142], they can also advance tumorigenesis, proliferation and drug resistance in some cases, such as promoting the progression of various sarcomas and maintaining leukemia-initiating cells (LISs), leading to drug resistance following human epidermal growth factor receptor 2 (HER2) inhibition in *HER2*(+) breast cancer [143]. In addition, FOXOs modulate metabolism and improve insulin resistance [143]. Current studies of *FOXOs* have focused on the mechanism underlying aging-related diseases, and some *FOXO* knockout mouse models are under development [144, 145].

*SIRT.* Seven *SIRT* genes (*SIRT1-7*) are currently known in humans. Their encoded sirtuin protein family is a relatively clear factor negatively related to aging, and its function was first demonstrated in yeast. In mammals, the sirtuin protein family is a group of class III histone deacetylases (HDACs) homologous to the silent information regulator 2 (Sir2) protein in yeast. These genes encode proteins with different functions, substrate affinities and subcellular compartmentalization. However, all these proteins share similar catalytic domains and rely on NAD+ as a co-reactive substrate to regulate the epigenetic modification of genes [146]. Currently, these seven proteins have been studied to various extents. SIRT1 is the most well-studied. It plays a crucial role in the mitochondrial signaling pathway, chronic inflammation, metabolism and interaction with multiple other signal pathways including NF-κB, AMPK, mTOR, P53, PGC1α, and FOXOs [147]. SIRT6 is under the spotlight for aging as it regulates genome and epigenome stability [148]. SIRT4 participates in the maintenance of glutamine catabolism and adenosine triphosphate (ATP) homeostasis [149]. SIRT7, the most recently identified mammalian sirtuin, has been reported to regulate the DNA damage response and DNA repair [150]. In contrast, the functions of SIRT2, SIRT3 and SIRT5 remain not entirely clear. Studies of SIRT are shifting from familiarity with signaling pathways to its utilization in mammalian models of neurodegeneration, nephropathy, cardiovascular disease, and metabolic-related diseases [8, 151-155]; and some of these proteins displayed antitumor effects [152]. Correspondingly, some drugs for regulating these signaling pathways, such as SRT2104 (a selective small molecule activator of SIRT 1) [156] and MIB-626 (a form of nicotinamide mononucleotide (NMN)) that increase NAD^+^ levels [157], have emerged and been evaluated in clinical trials. Among the abovementioned diseases, SIRT is widely believed to be the most closely associated with cardiovascular and metabolic diseases. Therefore, gene therapy with *SIRT* begins with these diseases and is being developed. Sun et al. generated a conditional progerin (*Lmna*^G609G^) knock-in model induced by Tie2-Cre for Hutchinson-Gilford progeria syndrome (HGPS) [158]. They observed a SIRT7-related defective microvasculature and inflammatory response and alleviated the disease phenotype as well as extended the lifespan in progeria mice by delivering *SIRT7 via* AAV1 (Table 2). In addition, Han et al. analyzed data from the Oncomine database and clinical samples, confirming *SIRT6* expression and its potential mechanism in prostate cancer progression. Eventually, they silenced SIRT6 by engineered exosomes that delivered siRNA to efficiently suppress tumor growth and metastasis on xenografted tumor models of mice [159]. Further research is expected to continue *in vivo*.

*VEGF.* The entire human body is widely affected by vascular endothelial growth factor (VEGF), and the formation and function of blood vessels are highly reliant on VEGF. VEGF is negatively associated with aging, and its high VEGF expression has a protective effect on the cardiovascular system [160]. However, many studies have identified it as a potential target in malignant tumors and it is regarded as a promoting factor [161]. Therefore, it remains a concern whether it will induce cancer when applied in anti-aging gene therapy. Grunewald et al. conducted an experiment based on the hypothesis that vascular aging is a founding factor in organismal aging. Over an experimental period of more than 30 months, they reported increased lifespans and physiological function after applying the gain-of-function system of transgenic *VEGF* and AAV-assisted *VEGF* transduction. And they revealed the mechanism by which *VEGF* influences multiple organ systems (by age-related increases in decoy receptors), supporting the initial hypothesis [162] (Table 2). Thus, *VEGF* seems to play a paradoxical role in aging similar to the telomerase gene, which inspires further systematic and careful exploration of potential treatments.

## Safety and feasibility of future applications

Gene therapy has been controversial since it was first proposed. Currently, the question has changed from whether human gene therapy should be developed to how it should be developed, supervised, and yield preliminary success. On September 13, 1999, 18-year-old Jesse Gelsinger died of multiple organ failure 4 days after receiving an injection of an adenovirus carrier. This event was presumably due to an excessive immune reaction to the adenovirus carrier but prompted in-depth and systematic exploration of gene therapy as well as an adequate understanding of its key issues. Thus, successive strategies that apply updated gene therapy products have continuously improved their safety and feasibility. Currently, the rAAV-mediated gene replacement strategy appears best positioned to translate into clinical applications for aging-related diseases. This method does not alter the sequence and structure of the genome, thus preventing the introduction of genome instability factors and enhancing safety. Therefore, rAAV-mediated gene replacement has been widely used in clinical trials involving a variety of gene therapies, including but not limited to aging. Moreover, abundant and emerging rAAV serotypes allow targeting of anatomically and functionally distinct parts of the body. Although LNPs and other nonviral vehicles also possess good safety and are likely to be widely used in clinical practice, they are still being developed for higher tissue or organ specificity, and elaborate designs are required to achieve better feasibility. To address different needs, standardized procedures can be implemented to regulate treatment quality. Thus, rAAV-mediated gene replacement has wider application prospects. Current examples of this strategy include Zolgensma® for spinal muscular atrophy and Luxturna® for RPE65-related retinal dystrophy, both of which are approved for listing, illustrating the safety and feasibility of this strategy for future applications.

## Conclusion and perspective

Gene therapies, especially in the field of aging provide new hopes for treating diseases. However, not all aging-related genes have the potential to be the targets. Satisfactory efficacy is only achieved by combining them with an adaptive operational strategy and efficient carriers. Additionally, some genes may simply be predictors of prognosis or capable of screening out effective medications for diseases[163-166]. To be a therapeutic target, the candidate gene should have a relatively distinct and clear role. And it is imperative to have a good synergy with other genes and signaling pathways to avoid subsequent excess signaling or signaling cascades [44, 107], which result in uncontrollable adverse reactions. Although a large number of gene therapies have been effective in mammalian models, the most utilized strategy is still gene replacement therapy with rAAV vectors. Advances in strategies and products of gene therapy will increase its efficacy and reduce adverse reactions. We anticipate the emergence of more trials and products over time. In addition, the seeming paradox of the involvement of the telomerase gene and *VEGF* in aging provides a better understanding of targetability and holistic perspective. Future research should focus on selecting and applying aging-related genes, developing optimal methods of treatment, and improving targetability, reproducibility and efficiency in clinical practice. While translational applications from the bench to the bedside may be slow, the patients suffering from age-related diseases may be rejuvenated eventually.
